# Therapeutic Efficacy of Cintredekin Besudotox (IL13-PE38QQR) in Murine Lung Fibrosis Is Unaffected by Immunity to *Pseudomonas aeruginosa* Exotoxin A

**DOI:** 10.1371/journal.pone.0008721

**Published:** 2010-01-15

**Authors:** Rogério S. Rosada, Ana P. Moreira, Fabiani G. Frantz, Raj K. Puri, Aquilur Rahman, Theodore J. Standiford, Carlos R. Zárate-Bladés, Célio L. Silva, Cory M. Hogaboam

**Affiliations:** 1 Núcleo de Pesquisa em Tuberculose, Departamento de Bioquímica e Imunologia, Universidade de São Paulo, São Paulo, Brazil; 2 Department of Pathology, University of Michigan Medical School, Ann Arbor, Michigan, United States of America; 3 Division of Pulmonary and Critical Care Medicine, University of Michigan Medical School, Ann Arbor, Michigan, United States of America; 4 Tumor Vaccines and Biotechnology Branch, Division of Cellular and Gene Therapies, Food and Drug Administration, Bethesda, Maryland, United States of America; 5 NeoPharm Inc., Lake Bluff, Illinois, United States of America; Emory University, United States of America

## Abstract

**Background:**

We have previously explored a therapeutic strategy for specifically targeting the profibrotic activity of IL-13 during experimental pulmonary fibrosis using a fusion protein comprised of human IL-13 and a mutated form of *Pseudomonas aeruginosa* exotoxin A (IL13-PE) and observed that the intranasal delivery of IL13-PE reduced bleomycin-induced pulmonary fibrosis through its elimination of IL-13-responsive cells in the lung. The aim of the present study was to determine whether the presence of an immune response to *P. aeruginosa* and/or its exotoxin A (PE) would diminish the anti-fibrotic properties of IL13-PE.

**Methodology/Principal Findings:**

Fourteen days after *P. aeruginosa* infection, C57BL/6 mice were injected with bleomycin via the intratracheal route. Other groups of mice received 4 doses of saline or IL13-PE by either intranasal or intraperitoneal application, and were challenged i.t. with bleomycin 28 days later. At day 21 after bleomycin, all mice received either saline vehicle or IL13-PE by the intranasal route and histopatological analyses of whole lung samples were performed at day 28 after bleomycin. Intrapulmonary *P. aeruginosa* infection promoted a neutralizing IgG2A and IgA antibody response in BALF and serum. Surprisingly, histological analysis showed that a prior *P. aeruginosa* infection attenuated the development of bleomycin-induced pulmonary fibrosis, which was modestly further attenuated by the intranasal administration of IL13-PE. Although prior intranasal administration of IL13-PE failed to elicit an antibody response, the systemic administration of IL13-PE induced a strong neutralizing antibody response. However, the prior systemic sensitization of mice with IL13-PE did not inhibit the anti-fibrotic effect of IL13-PE in fibrotic mice.

**Conclusions:**

Thus, IL13-PE therapy in pulmonary fibrosis works regardless of the presence of a humoral immune response to *Pseudomonas* exotoxin A. Interestingly, a prior infection with *P. aeruginosa* markedly attenuated the pulmonary fibrotic response suggesting that the immune elicitation by this pathogen exerts anti-fibrotic effects.

## Introduction

Idiopathic pulmonary fibrosis (IPF) is a fatal, interstitial lung disease characterized by persistent tissue scarring for which there is no effective therapy. The diagnostic lesion of IPF is the fibroblastic foci comprised of a heterogeneous mix of epithelial cells and fibroblasts, which, it is postulated, forms as a result of an inappropriate wound healing response to an unknown injurious agent [1]. Since the overall cytokine pattern in biopsies and alveolar macrophages from patients with interstitial pneumonia appears to be more Th2-type (i.e., IL-4 and IL-13) than Th1-type (i.e., IL-12 and IFN-γ) [2], [3], [4], [5], a highly anticipated antifibrotic strategy within the lung entails the targeted inhibition of both IL-4 and IL-13. Although transgenic over-expression of IL-13 alone in the lung leads to the development of pulmonary fibrosis [6], [7], both IL-4 and IL-13 appear to contribute to the development of pulmonary fibrosis [8], [9], presumably due to their ability to act directly on pulmonary fibroblasts [10] and mononuclear cells/macrophages [11]. IL-4Rα and IL-13Rα1 form a functional receptor complex that binds both ligands [12], [13]. IL-13, but not IL-4 [14], also binds with 100-fold higher affinity for IL-13Rα2 than IL-13Rα1 [15]. IL-13R subunits are expressed on a variety of immune and nonimmune cells, including B cells, NK cells, monocytes, mast cells, endothelial cells, and fibroblasts [10], [12], [16], [17], [18].

A therapeutic strategy for specifically targeting the profibrotic activity of IL-13 in the lung involves a fusion protein comprised of human IL-13, which binds to mouse receptors and a mutated form of *Pseudomonas aeruginosa* exotoxin A (Cintredekin Besudotox, IL-13-PE38QQR, or IL13-PE) [19]. IL13-PE was initially developed to selectively target and kill tumor cells with abnormal responses to IL-13 due to markedly up-regulated expression of IL-4R and IL-13R [19], [20]. We demonstrated that the intranasal delivery of IL13-PE significantly reduced *Aspergillus fumigatus*-induced peribronchial [21], [22], *Schistosoma mansoni*-induced granulomatous [23], and bleomycin-induced [24] fibrosis *in vivo* through its reduction in the number of IL-13-responsive immune and resident lung cells such as macrophages, eosinophils, NK cells, and fibroblasts. Previous studies have documented that IL-13 is also elevated during the pulmonary response to an intrapulmonary bleomycin sulfate challenge [11], [25], inducing alveolar interstitial inflammation that precedes an exuberant and inappropriate tissue repair response in the lung [26], [27].

Because the existence of neutralizing antibodies directed against *Pseudomonas* exotoxin A could potentially reduce the therapeutic effects of IL13-PE in the fibrotic lung, we examined whether the existence of an immune response due to prior *Pseudomonas aeruginosa* infection or sensitization to IL13-PE might diminish or abolish the anti-fibrotic effects of IL13-PE. To this end, we addressed the following three questions: 1. Does an intrapulmonary *P. aeruginosa* infection promote a neutralizing antibody response in the lung? 2. Does prior pulmonary exposure to *P. aeruginosa* infection modulate the therapeutic effects of IL13-PE? 3. Do circulating IL13-PE-specific antibodies neutralize the therapeutic effects of intranasally delivered IL13-PE during pulmonary fibrosis? Overall, we found that despite the strong immunogencity of an active infection with *P. aeruginosa* or systemic sensitization with IL13-PE, the intranasal delivery of IL13-PE robustly inhibited bleomycin-induced pulmonary fibrosis. Taken together, our results suggest that prior patient exposure to *Pseudomonas* or immunity directed against its exotoxin does not diminish the therapeutic potential of IL13-PE in the treatment of pulmonary fibrosis.

## Results

### *Pseudomonas aeruginosa* Infection Induces both IgA and IgG2a Antibodies Directed against *Pseudomonas aeruginosa* Exotoxin A

**Figure 1:**
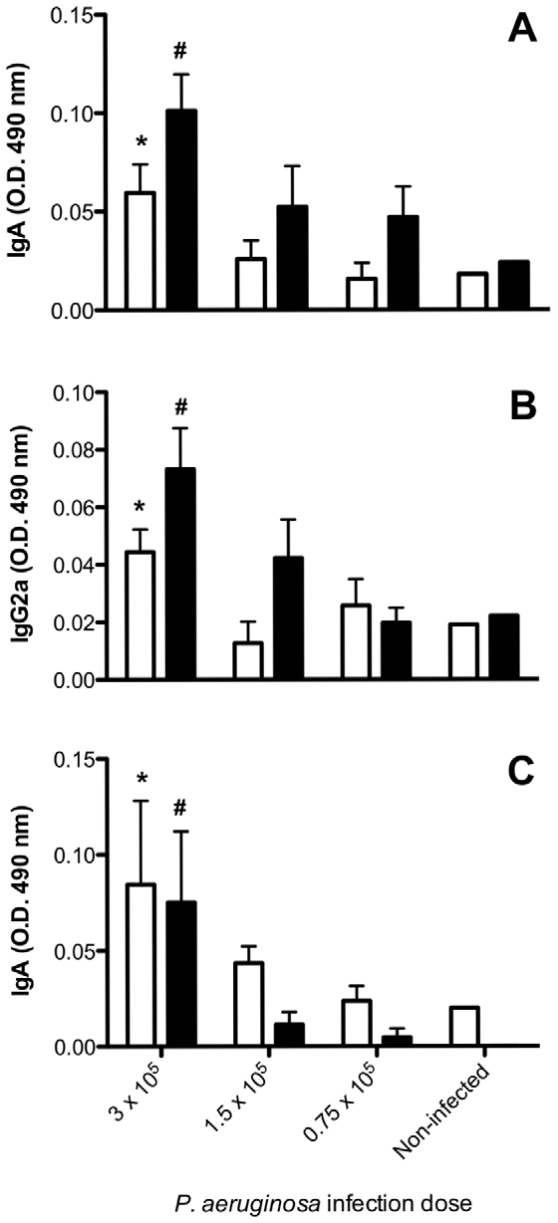
*P. aeruginosa* infection induced an antibody response against PE and IL13-PE. **A**: IgA levels in BAL; **B**: IgG2a in BAL; **C**: IgA in serum. C57Bl/6 mice were infected by oropharingeal route with one of a range of bacterial doses and their bronchoalveolar fluid (BAL) and blood were collected at 14 days post infection. Antibody levels were evaluated by ELISA using plates coated with PE (open bars) or IL13-PE (closed bars). Statistical comparisons were determined between infected and non-infected groups with the same coating on the ELISA plate. *p≤0.05 compared with biologic sample added to PE-coated wells. ^#^p≤0.05 compared with biologic sample added to IL13-PE-coated wells.

To evaluate the amount of bacilli necessary to provoke specific antibody production against PE, we infected mice with one of three non-lethal *P. aeruginosa* doses: the lowest of 0.75×10^5^ bacilli/mouse, an intermediate dose of 1.5×10^5^ bacilli/mouse and the highest does of 3×10^5^ bacilli/mouse. As shown in **Figure 1**, all three bacilli doses elicited detectable increases in IgA and IgG2a antibody titers 14 days after infection in BAL samples (**Panels A & B**, respectively) and IgA in serum (**Panel C**). The increases in both IgA and IgG2a were observed in ELISA plates coated either with purified PE toxin from *P. aeruginosa* extracts or coated with intact IL13-PE. Therefore, *P. aeruginosa* infection elicited strong humoral responses as evidenced by increased IgA and IgG2a.

### A Prior *P. aeruginosa* Infection Modulates the Subsequent Development of Pulmonary Fibrosis

**Figure 2:**
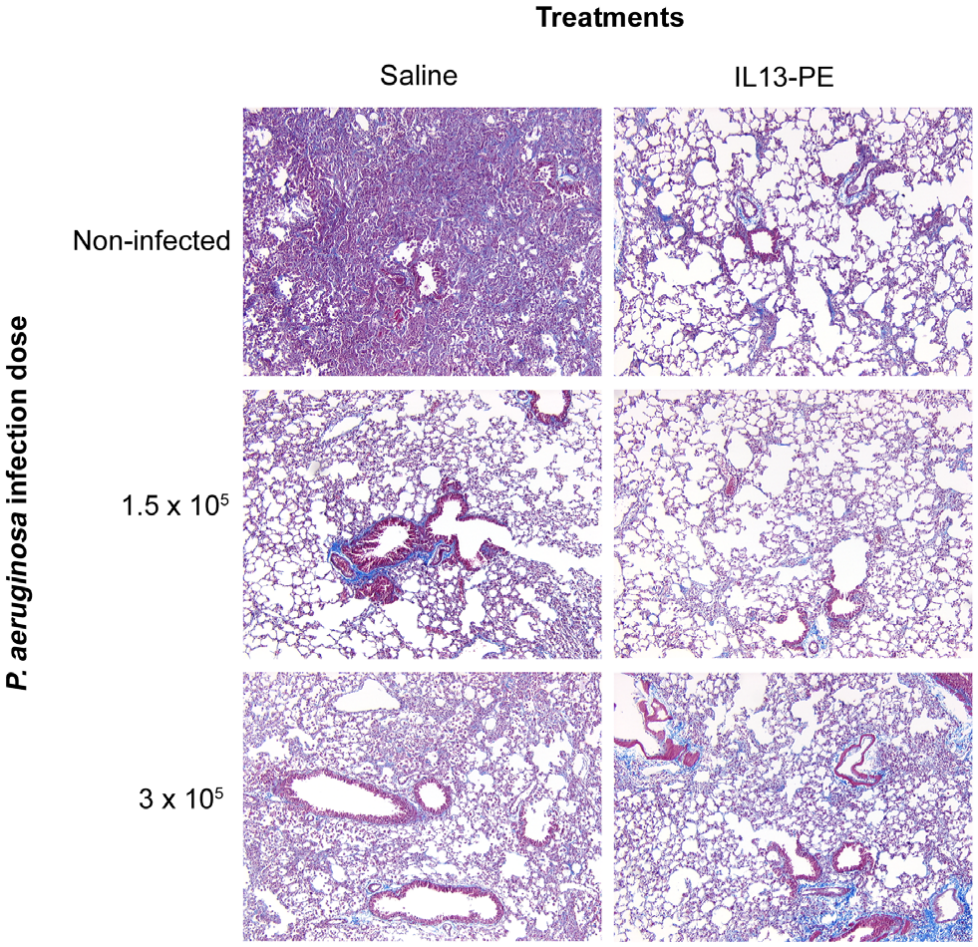
IL13-PE treatment reduced the histological appearance of pulmonary fibrosis regardless of the prior *P.* *aeruginosa* infection status of the mouse. C57Bl/6 mice were infected by oropharingeal route with two bacterial doses (1.5×10^5^ or 3×10^5^ bacilli/mouse), fourteen days after infection mice were injected with bleomycin to exacerbate fibrosis and at days 21, 23, 25 and 27 after bleomycin mice received intranasal IL13-PE treatments (1000 ng/dose). At day 28 after bleomycin, lungs were collected and processed using routine histological techniques and stained with Masson's trichrome for histopathological analysis. Magnification 100×.

Having shown that *P. aeruginosa* infection induces a specific antibody against PE, we next determined whether this prior infection altered the efficacy of IL13-PE therapy in a pulmonary fibrosis model induced by the intratracheal introduction of bleomycin sulfate. As we have observed previously [24], mice that received bleomycin developed severe pulmonary fibrosis (**Figure 2**) but the introduction of four intranasal doses of IL13-PE (1000 ng/dose between days 21 and 28 after bleomycin ameliorated this fibrotic response (**Figure 2**). We observed that the pulmonary fibrotic response elicited by bleomycin in mice challenged with *P. aeruginosa* prior to bleomycin instillation was markedly diminished compared with the uninfected control group. This protective effect was more dramatic in the group of mice that received 1.5×10^5^ bacilli compared with the group of mice that received 3×10^5^ bacilli (**Figure 2**). While the intranasal administration of IL13-PE appeared to reduce the extent of the fibrosis observed in these previously infected mice, the improvement was minor given their diminished fibrotic response to bleomycin. Thus, prior pulmonary exposure to live *Pseudomonas* bacilli appeared to protect mice from the fibrotic effects of bleomycin sulfate through as yet to be defined mechanism. IL13-PE therapy in these groups of mice further reduced the minor fibrotic response indicating that its activity was not diminished by the presence of neutralizing antibodies against PE.

### Systemic Sensitization to IL13-PE Generates Neutralizing Antibodies in Mice That Block the *In Vitro* Toxicity of IL13-PE Directed toward Tumor Cells

The infection with live pathogen addressed, in part, our concern regarding its impact on the therapeutic effectiveness of IL13-PE in pulmonary fibrosis. We next addressed the concern that local or systemic sensitization to IL13-PE would negate the therapeutic efficacy of IL13-PE in the fibrotic lung. Neutralizing antibody generation following sensitization was assessed using a well-described *in vitro* cytotoxicity assay [28] in which serum from our various treatment groups were added. We found that delivery of 4 doses of IL13-PE into the lungs of mice failed to elicit a neutralizing antibody response (not shown), but as shown in **Table 1** systemic sensitization with PE lead to the appearance of neutralizing antibodies in serum, which markedly inhibited the cytotoxicity of IL13-PE in this *in vitro* assay (i.e. the IC_50_ values were > 10 ng/ml). Thus, the systemic but not local delivery of IL13-PE led to the development of a neutralizing antibody response, which inhibited the *in vitro* toxicity of IL13-PE in IL-13-responsive tumor cells.

**Table 1:** IL13-PE cytotoxicity toward an IL-13Rα2-expressing tumor cell line in the presence of mouse sera from various groups of treated mice^a^.

Treatment	(IC_50_ ng/ml)^b^	
	Saline	IL13-PE Sensitized
Saline	0.85	>10/>10/>10
IL13-PE (200 ng/dose)	0.6/0.4	>10/>10
IL13-PE (500 ng/dose)	0.5	>10/>10
IL13-PE (1000 ng/dose)	0.4/0.5	>10/>10/>10
Pre-bleomycin sample	0.5	>10

a Mice were systemically sensitized to IL13-PE as described in the Materials and Methods section. Prior to bleomycin challenge, blood samples were removed from the control (saline) and IL13-PE-treated groups (ie. the pre-bleomyin sample). Mice received bleomycin and 21 days later they were randomized to treatment groups, which received one of saline alone, 200 ng/dose of IL13-PE, 500 ng/dose of IL13-PE, or 1000 ng/dose of IL13-PE. Sera was removed from each group at day 28 after 4 treatments of saline or IL13-PE. For this cytotoxicity assay, 1×10^4^ tumor cells were cultured with IL13-PE and serum sample for 20 hr at 37°C, pulsed with 1 µCi of [^3^H]-leucine and further incubated for 4 hr. Cells were harvested and counted as described in the Materials and Methods section.

b IC_50_ is the concentration of IL13-PE at which a 50% inhibition of protein synthesis occurs in IL13-PE treated tumor cells compared with untreated tumor cells.

### Systemic Sensitization to IL13-PE Did Not Alter the Therapeutic Efficacy of IL13-PE in Experimental Pulmonary Fibrosis

**Figure 3:**
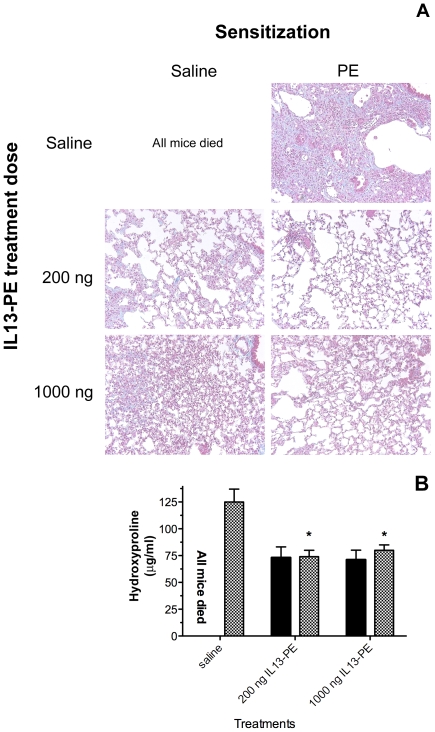
Effect of systemic IL13-PE sensitization on the development of pulmonary fibrosis and the subsequent intranasal IL13-PE therapy. Mice were sensitized with IL13-PE via an intraperitoneal immunization protocol. Controls received saline alone. One week after last dose of IL13-PE or saline, all mice were injected i.t. with bleomycin. Twenty-one days after the induction of pulmonary fibrosis, treatment started with saline or IL13-PE intranasal instillation. At day 28 after bleomycin, lungs were collected for histopathological or biochemical assessment of pulmonary fibrosis. Magnification 100× (**A**). Hydroxyproline content in lung homogenate from saline (closed bars) or IL13-PE (hatched bars) sensitized animals are shown (**B**).

Given that the systemic sensitization to IL13-PE resulted in appearance of neutralizing antibodies in the serum, we next evaluated the influence of these antibodies on the therapeutic effect of IL13-PE against pulmonary fibrosis. In this experiment, all of the mice receiving bleomycin alone were dead by the day 28 timepoint due to an aggressive pulmonary fibrotic response. Prior sensitization to IL13-PE protected bleomycin-challenged mice from mortality but not from pulmonary fibrosis (**Figure 3** **, panel A, saline + PE group**). More importantly, regardless of the IL13-PE sensitization status of the mouse prior to bleomycin challenge, IL13-PE therapy markedly reduced the histological appearance of fibrosis (**Figure 3** **, Panel A**). Specifically, lungs from mice that received saline showed a marked reduction in lung fibrosis after intranasal treatment with either 200 or 1000 ng/dose of IL13-PE (Figure 3, Panel A, left side of Figure). Likewise, the intranasal administration of either dose of IL13-PE markedly suppressed the interstitial fibrosis in PE-sensitized mice (Figure 3, Panel A, right side of Figure). These histological findings were confirmed with the quantitative assessment of hydroxyproline content (**Figure 3** **, panel B**). Thus, these findings demonstrate that the systemic sensitization to IL13-PE does not predispose mice to the development of a more aggressive form of fibrosis nor does it prevent the intranasal delivery of IL13-PE from exerting a prominent anti-fibrotic effect.

## Discussion

The bleomycin model is one of the best-characterized models of fibrosis since this chemotherapeutic invokes a highly reproducible inflammatory response that ultimately leads to fibroblast proliferation and collagen deposition [29], [30]. IL-13 is one of the dominant pro-fibrotic cytokines produced during the development of fibrosis in the bleomycin model since it directly activates fibroblasts [30]. Puri and colleagues [31], [32] have previously shown that tumor cells expressing IL-13Rα2 are susceptible to the cytotoxic effects of IL13-PE making this chimeric protein a highly effective anti-tumor agent, and we have extended these findings to show that targeting IL-13 responsive cells such as fibroblasts is also highly effective in treating established fibrotic responses in the lung [24]. Prior to further development of IL13-PE as a therapeutic in clinical disease we undertook the present study in order to evaluate whether prior exposure to *P. aeruginosa* pathogen or IL13-PE altered the therapeutic potential of IL-13 immunotoxin. Our study highlighted two important findings: 1) immune responses to *Pseudomonas* and/or its exotoxin actually protect mice from bleomycin-induced pulmonary fibrosis; 2) neutralizing antibodies against *Pseudomonas* and/or its exotoxin do not hamper the therapeutic effect of IL13-PE delivered into the fibrotic lung.

*P. aeruginosa* is a pathogen of significant clinical importance because it can elicit a severe pneumonia in affected individuals. No prior association between this pathogen and fibrosis was apparent in our literature searches so we undertook experiments to better understand how this micro-organism might affect the lung subsequently exposed to bleomycin sulfate. While antibody responses to live *Pseudomonas* infections in the lung have been described previously [33], [34], little was known about the specificity of these antibody responses against *Pseudomonas* exotoxin. From the present study it was apparent that relatively small intrapulmonary inoculations of live *Pseudomonas* bacilli evoked strong IgA and IgG2a anti-exotoxin antibody systemic (in serum) and local (in lung) responses. When we next examined the impact of a prior *P. aeruginosa* infection on the susceptibility of the lung to fibrosis, we were surprised to observe that prior infection protected mice from severe pulmonary fibrosis. Protection from fibrosis did not correlate with the retention of bacilli in the lungs of these mice as no bacteria were recovered from these mice prior to bleomycin injection day (data not show). These findings were surprising in light of several studies that have shown that repeated exposure or chronic infections with pathogens such as *Paracoccidioides brasiliensis* [35], [36], *Saccharopolyspora rectivirgula* [37] and many others [38] leads to the development of pulmonary fibrosis. The divergence observed between the present and previous studies might be explained by the rapid clearance of *P. aeruginosa* in the present study prior to the induction of pulmonary fibrosis with bleomycin. It is possible that acute bacterial infection drives a protective immune response that tempers bleomycin-induced fibrosis. We are presently addressing the hypothesis that this protective effect observed in acutely infected mice involves pathogen-associated molecular pattern (PAMP) activation of toll like receptors (TLRs). Specifically, we have observed that PAMPS such as bacterial hypomethylated DNA or CpG motifs inhibit the development and progression of bleomcyin-induced pulmonary fibrosis (CMH, unpublished findings). When these CpG motifs bind TLR9 in lung immune cells, this leads to the generation of type 1 interferons, which have potent anti-fibrotic effects. Further studies will address the possibility that CpG and TLR9 activation in immune cells accounts, at least in part, for the protective effects of a prior *P. aeruginosa* infection.

Systemic but not local IL13-PE dosing led to the development of neutralizing antibodies against this chimeric protein, which effectively blocked the cytotoxic action of IL13-PE *in vitro* toward tumor cells. Using a well-characterized tumor toxicity assay [28], [39], we analyzed the ability of various serum samples from mice systemically sensitized to IL13-PE to block the cytotoxicity of IL13-PE against IL-13Rα2-expressing tumor cells. We observed that serum from IL13-PE-sensitized mice markedly dampened the IL13-PE-induced toxicity against target tumor cells suggesting the presence of neutralizing antibodies directed against IL13-PE in serum. Accordingly, the development of neutralizing antibodies to IL13-PE was a concern given that these neutralizing antibodies might interfere with IL13-PE therapy in pulmonary fibrosis. However, despite the presence of the neutralizing antibodies in sensitized mice, intranasal IL13-PE therapy worked effectively as a therapeutic in the bleomycin-induced pulmonary fibrosis model. The manner in which IL13-PE continues to work in the lung despite the presence of these neutralizing antibodies is not presently clear but we have observed that the numbers of IL-13Rα2-positive cells during pulmonary fibrosis are markedly increased ([24] and unpublished findings). Under these conditions it is possible that the neutralizing titers of antibody are not sufficient to prevent IL13-PE from binding to this high affinity IL-13 receptor. Further studies will address this possibility in the lung.

Thus, using a well-established bleomycin pulmonary fibrosis model, we have observed that prior *Pseudmonas* infection or systemic sensitization to IL13-PE does not impair the therapeutic anti-fibrotic properties of IL13-PE. Surprisingly, both of these events provide protective effects either by reducing the fibrotic response (as in the case of infection) or sparing mice from the lethal effects of pulmonary fibrosis (as in the case of systemic IL13-PE sensitization). Together, these findings further bolster the prospects of IL13-PE as a clinically useful therapeutic in the treatment of pulmonary fibrosis.

## Materials and Methods

### Animals

Female C57Bl/6 mice (6- to 8-week-old) were purchased from Taconic Farms (Germantown, NY). All mice were maintained in specific pathogen-free conditions and provided with food and water *ad libitum*. Prior approval to conduct these studies was obtained from an University Committee on the Use and Care of Animals at the University of Michigan Medical School.

### Pulmonary *P. aeruginosa* Infection

*P. aeruginosa* was grown as previously described [40]. Briefly, a 1∶1,000 dilution of *P. aeruginosa* stock was grown in tryptic soy broth (TSB) (DIFCO, Detroit, MI) for 18 hr at 37°C. Bacterial concentration was determined by measuring absorbance at 600 nm compared with a predetermined standard curve. Bacteria were diluted to the desired concentration for inoculation. Mice were anaesthetized by i.p. injection of mixture of 2.25 mg of ketamine hydrochloric acid (Abbott Laboratories, Chicago, IL) and 150 µg of xylazine (Lloyd Laboratories, Shenandoah, IA) and 30 µl of bacterial suspension was administered by oropharyngeal aspiration into lungs, as described by Lakatos and colleagues [41], at a range of bacterial doses: the lowest (0.75×10^5^ bacilli/mouse), intermediate (1.5×10^5^ bacilli/mouse) and highest dose (3×10^5^ bacilli/mouse).

### Bronchoalveolar Lavage Fluid (BALF)

Animals were killed with ketamine/xylazine overdose at appropriate times, the anterior chest cavity of each animal was carefully opened, and the trachea was exposed and catheterized. The catheter was tied in place and 0.5 ml of sterile PBS was infused. Lavage fluid was recovered and frozen until the determination of antibody titers.

### Antibody Titers Determination

Antibody titers in serum and BAL fluid were determined using an enzyme-linked immunosorbent assay (ELISA) using 96-well plates coated with purified PE toxin from *P. aeruginosa* extracts or with IL13-PE, both at a concentration of 5 µg/mL (50 µl/well, overnight incubations at 4°C). Then, wells were treated with blocking buffer, washed and samples were serially diluted in blocking buffer and incubated 2 hours at 37°C. Bound antibodies were detected using rabbit anti-mouse immunoglobulin A (IgA) or G (IgG2a) diluted appropriately in blocking buffer. A secondary biotinilated antibody was added and after avidin-peroxidase ligation, we added 100 µl of Substrate Solution (BD Pharmingen™ TMB SubstrateReagent Set) to each well and incubated the plates for 5 minutes at room temperature in the dark. The reaction was stopped by the addition of 50 µl of Stop Solution (2 N H_2_SO_4_) to each well and the optical density was measured at 450 nm within 30 minutes of stopping reaction.

### Bleomycin Model

Interstitial pulmonary fibrosis was induced in anaesthetized mice (2.25 mg of ketamine and 150 µg of xylazine) by the i.t. injection of 1.7 U/kg of mouse body weight of bleomycin (Blenoxane, sterile bleomycin sulfate; Bristol-Meyers Pharmaceuticals, Evansville, IN) dissolved in 60 µl of PBS as previously described in detail [26], [27]. Controls received 60 µl of PBS by the same route. All procedures were conducted in a sterile environment and were approved by the institutional animal care and use committee.

### Intranasal IL13-PE Therapy after Bleomycin Challenge in Mice

Fourteen days after *P. aeruginosa* infection mice were injected via the i.t. route with bleomycin to exacerbate fibrosis and at days 21, 23, 25 and 27 after bleomycin injection each mouse received 1000 ng/dose of IL13-PE in 30 µl of PBS by the intranasal route. At day 28, lungs were collected and analyzed as described below.

### Systemic Sensitization with IL13-PE and Subsequent Fibrosis Treatment with IL13-PE

Mice received either saline alone or saline with 50 mg/kg of IL13-PE by i.p. injection on 4 consecutive days. One week after the last injection, the appropriate group of mice received either saline or 50 mg/kg of IL13-PE by i.p. injection. One week following the boost, mice received the second boost of either saline or IL13-PE (50 mg/kg). Within hours of the second boost, blood was removed for the determination of the antibody titer in all mice and bleomycin was then administered as described above. At day 21 after bleomycin challenge, mice in both sensitization groups were divided into the following treatment groups: saline alone, saline +200 ng/dose, 500 ng/dose, and 1000 ng/dose of IL13-PE. Saline or IL13-PE was given by intranasal administration at days 21, 23, 25, and 27 after bleomycin challenge. All mice were killed at day 28 after bleomycin and serum were collected for neutralizing antibodies evaluation, and whole lungs were removed for hydroxyproline and histological analysis.

### Protein Synthesis Inhibition Assay

The cytotoxic activity of IL13-PE was tested as previously described by determining inhibition of protein synthesis [42]. Briefly, 10^4^ tumor cells were cultured in leucine-free medium with or without serum samples and various concentrations of IL13-PE for 20–22 hr at 37°C. Then, 1 µCi of [^3^H]-leucine (NEN Research Products, Wilmington, DE) was added to each well and cells were incubated for an additional 4 hr. Cells were harvested and radioactivity incorporated into cells was measured by a Beta plate counter (Wallac, Gaithersburg,MD).

### Hydroxyproline Assay

Total lung hydroxyproline levels were determined in saline or IL13-PE-treated mice following bleomycin challenge using a previously described assay [43]. Briefly, lungs were homogenated in 1 ml and 500 µl was collected and added to 1 ml of 6 N HCl for 8 hours at 120°C. To a 5-µl sample of the digested lung, 5 µl of citrate/acetate buffer (5% citric acid, 7.2% sodium acetate, 3.4% sodium hydroxide, and 1.2% glacial acetic acid, pH 6.0) and 100 µl of chloramine-T solution (282 mg chloramine-T, 2 ml of *n*-propanol, 2 ml of distilled water, and 16 ml of citrate/acetate buffer) were added. The resulting samples were then incubated at room temperature for 20 minutes and 100 µl of Ehrlich's solution (Aldrich, Milwaukee, WI), 9.3 ml of *n*-propanol, and 3.9 ml of 70% perchloric acid were added (Aldrich). These samples were incubated for 15 minutes at 65°C and cooled samples were read at 550 nm in a Beckman DU 640 spectrophotometer. Hydroxyproline concentrations were calculated from a hydroxyproline standard curve (0 to 100 µg of hydroxyproline/ml).

### Lung Histological Analysis

Whole left lobes of lungs were fully inflated with 10% formalin, dissected, and placed in fresh formalin for an additional 24 hours. Routine histological techniques were used to paraffin-embed the entire lung, and 5-µm sections of whole lung were stained with hematoxylin and eosin (H&E) or with Masson's trichrome.

### Statistical Analysis

All results are expressed as the mean ± SD. Student's *t* test were used to detect statistical differences between the control and treatment groups; *p*≤0.05 was considered statistically significant. GraphPad Prism version 5.0b was used for statistical analyses.
